# Kratom Toxicity: Pharmacologic Mechanisms, Clinical Risks, and Management Considerations

**DOI:** 10.7759/cureus.113280

**Published:** 2026-07-24

**Authors:** Asis A Babun, Brehyn R Evans, Ines N Volic, Sierra Wood, Kelson Knighton, Felipe Lopez, Patrick Tufts

**Affiliations:** 1 Military Medicine, Rocky Vista University College of Osteopathic Medicine, Ivins, USA; 2 College of Medicine, Rocky Vista University College of Osteopathic Medicine, Ivins, USA; 3 Family Medicine, Rocky Vista University College of Osteopathic Medicine, Ivins, USA; 4 Biomedical Sciences, Rocky Vista University College of Osteopathic Medicine, Ivins, USA; 5 Military Medicine, Noorda College of Osteopathic Medicine, Provo, USA; 6 Primary Care, Rocky Vista University College of Osteopathic Medicine, Ivins, USA

**Keywords:** 7‑hydroxymitragynine, emergency medicine, kratom, mitragyna speciosa, mitragynine, naloxone, overdose, polysubstance use, respiratory depression, toxicology

## Abstract

Kratom (*Mitragyna speciosa*) is an opioid-like botanical increasingly used for self-management of pain and opioid withdrawal and has been increasingly implicated in emergency department presentations for acute toxicity. Despite its growing use, kratom remains poorly understood in clinical practice, particularly with respect to its complex, metabolite-dependent pharmacology. Product heterogeneity, variable alkaloid composition, and frequent polysubstance use further complicate clinical recognition and management, raising important patient safety concerns. This narrative review synthesizes the published clinical, toxicological, and pharmacologic literature on kratom toxicity, including case reports, case series, observational studies, and prior reviews, with particular emphasis on respiratory depression, naloxone responsiveness, and post-reversal management considerations.

Available evidence suggests that kratom-associated toxicity can produce opioid-like toxidromes with variable responsiveness to naloxone. Reported cases demonstrate heterogeneity in clinical presentation and reversal, likely reflecting differences in alkaloid composition, metabolite activity, product variability, and co-ingestant exposure. Published reports also describe delayed or recurrent respiratory depression following initial naloxone response, creating challenges regarding appropriate observation periods and disposition decisions. These risks may be further amplified by polysubstance use and variability among commercially available kratom preparations. Kratom-associated toxicity represents an evolving patient safety challenge in acute care settings. Clinicians should recognize the potential for delayed or recurrent respiratory depression following naloxone administration and consider extended observation in selected patients to reduce preventable morbidity.

## Introduction and background

Kratom (*Mitragyna speciosa*), a tropical tree native to Southeast Asia, has traditionally been used for its stimulant and analgesic properties [1]. In recent years, it has gained popularity in the West as an alternative treatment for anxiety, pain, and opioid withdrawal due to its active alkaloids, mitragynine and 7-hydroxymitragynine, which interact with opioid receptors [1-3].

Despite its increasing use, kratom remains poorly understood in clinical practice, particularly with respect to its pharmacology, safety profile, and toxicity. The growing availability of kratom through online retailers and over-the-counter products has heightened the need for a better understanding of its pharmacologic effects, adverse events, and potential risks [3]. Improved knowledge of these factors is essential to inform evidence-based clinical practice, regulatory policies, and public health initiatives [4].

Given the increasing prevalence of kratom use and the absence of standardized detection and management guidelines, this narrative review synthesizes current clinical, toxicologic, pharmacologic, and epidemiologic evidence regarding kratom toxicity and its implications for acute care. Particular emphasis is placed on respiratory depression, variable responsiveness to naloxone, delayed or recurrent respiratory depression, and practical considerations for patient monitoring and disposition. In addition, this review discusses the evolving regulatory landscape and highlights implications for patient care and public health policy.

## Review

Materials and methods

A literature search was conducted using PubMed, Google Scholar, Embase, Scopus, and the Cochrane Library to identify publications relevant to kratom pharmacology, toxicology, clinical manifestations, and management. Search terms included combinations of "kratom," "Mitragyna speciosa," "mitragynine," "7-hydroxymitragynine," "toxicity," "overdose," "poisoning," "respiratory depression," "naloxone," and "opioid." A broad range of study designs was included, encompassing experimental pharmacologic studies, toxicology surveillance reports, observational studies, case reports, and narrative and systematic reviews. Reference lists of selected articles were manually reviewed to identify additional relevant publications.

Given the narrative and hypothesis-generating nature of this review, no formal inclusion or exclusion criteria were applied. Priority was given to peer-reviewed human studies, well-documented case reports, and toxicologic data with relevance to emergency and inpatient care. This review was not intended to be systematic and did not follow the Preferred Reporting Items for Systematic Reviews and Meta-Analyses (PRISMA) guidelines. Rather, it was designed to synthesize emerging clinical patterns and management challenges described in the literature rather than establish treatment protocols or causal relationships.

Overview of kratom

Kratom use differs markedly between Southeast Asia and the West, not only in cultural context but also in how the substance is prepared and its resulting effects. In Southeast Asia, kratom is traditionally consumed by chewing fresh leaves or brewing them into tea shortly after harvest - methods that preserve lower levels of 7-hydroxymitragynine, a potent opioid-like alkaloid. This long-standing practice, especially among laborers seeking energy and pain relief, has been associated with few documented reports of serious adverse events [1].

In contrast to its traditional use, kratom products in the West are typically derived from dried and processed leaves and are formulated into powders, capsules, or extracts, which can concentrate active alkaloids and facilitate higher total doses than traditional preparations. This processing may increase the risk of toxicity [5,6]. Western users often turn to kratom for self-managing chronic pain or opioid withdrawal [3].

The primary active compounds, mitragynine and 7-hydroxymitragynine, interact with μ-opioid receptors [1,2,7]. Mitragynine displays partial agonist-like effects in vivo but acts as a functional antagonist at human μ-opioid receptors in vitro [2,7]. In contrast, 7-hydroxymitragynine demonstrates greater efficacy, contributing to analgesic and sedative effects while also posing risks such as dependence and respiratory depression [1,2,7].

At lower doses, kratom has stimulant properties, whereas at higher doses it can induce sedation and opioid-like effects [1,6]. As global consumption rises, understanding these differences in preparation, pharmacokinetics, and safety profiles is essential for guiding public health policy and clinical practice [4].

Pharmacology and mechanisms of toxicity

**Figure 1:**
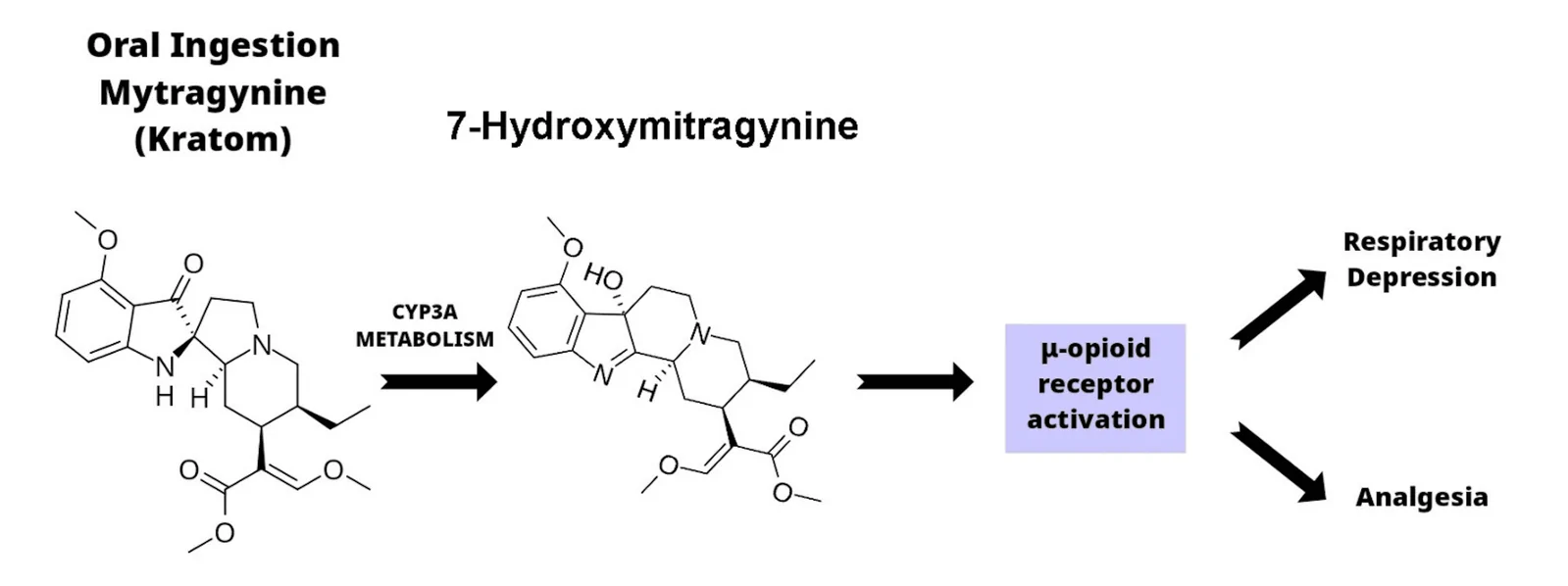
Proposed metabolic pathway linking mitragynine to opioid‑like effects Following oral ingestion, mitragynine undergoes CYP3A‑mediated metabolism to the active metabolite 7‑hydroxymitragynine, which exhibits greater μ‑opioid receptor agonism. Activation of μ‑opioid receptors is associated with analgesic effects and may contribute to respiratory depression at higher exposures or in susceptible individuals. This figure was created by the authors using CorelDRAW (Corel Corporation, Ottawa, ON, Canada) and reflects evidence synthesized from the preclinical and clinical literature.

Determining deaths caused solely by kratom presents significant challenges, as many individuals who consume kratom also use other opioids or psychoactive substances [8-10]. Kratom contains mitragynine, a partial μ-opioid receptor agonist. Experimental and pharmacologic studies indicate that opioid-like respiratory depression is more strongly associated with the activity of its metabolite, 7-hydroxymitragynine, which exhibits greater μ-opioid receptor efficacy (Figure 1) [2,11,12].

Naloxone, a μ-opioid receptor antagonist, is used to reverse opioid-induced respiratory depression. However, the duration of action of naloxone is substantially shorter than the reported half-life of mitragynine, kratom's principal alkaloid, which may increase the risk of recurrent respiratory depression with subsequent hypoxemia after initial reversal and pose important management challenges in emergency care settings [12-14]. Recent preclinical evidence further elucidates the mechanistic basis for variable responsiveness to naloxone in kratom toxicity. 7-hydroxymitragynine produces dose-dependent respiratory depression comparable to morphine and is reversed by naloxone in rats monitored with whole-body plethysmography, whereas mitragynine unexpectedly increases respiratory frequency and is not responsive to naloxone. These findings suggest that responsiveness to naloxone may depend on which alkaloid predominates in the consumed product [15].

An analysis of statewide mortality surveillance data from Florida between 2020 and 2021 showed that kratom is frequently detected in drug overdose deaths; however, kratom-only fatalities are rare, with the vast majority of cases involving one or more co-ingestants [8]. These findings highlight the frequent co-use of other substances and underscore the importance of understanding kratom's pharmacologic profile, as respiratory depression associated with kratom toxicity is linked to opioid receptor activity [1,2]. This risk may be amplified by the way kratom is processed into powders, extracts, and ethanol-based solutions for consumption in the United States [6].

Understanding kratom's dose-response profile is essential. At low doses, kratom acts as a stimulant, in part through adrenergic and serotonergic activity, whereas at higher doses it can produce sedation, respiratory depression, and, in rare cases, hepatotoxicity reported in the case literature [1,6]. Kratom also poses interaction risks through inhibition of cytochrome P450 enzymes (CYP3A4, CYP2D6, CYP2C9), which metabolize opioids, benzodiazepines, antidepressants, antipsychotics, and stimulants. This inhibition can elevate drug concentrations, intensify adverse effects, and prolong drug action, potentially leading to toxicity [11,16].

Mitragynine and 7-hydroxymitragynine both bind to μ-opioid receptors, with 7-hydroxymitragynine exhibiting greater agonist efficacy and producing more potent opioid-like effects [2,7,12]. Additionally, variability in kratom's chemical composition across products and preparations can influence its potency and risk, complicating dose regulation [5,16]. These pharmacologic characteristics are important considerations for clinicians managing kratom toxicity and for policymakers developing evidence-based public health regulations [4].

Current state laws regarding kratom

As of 2025, more than 20 states in the United States have enacted laws or introduced legislation regulating kratom [17]. Most of these regulations focus on consumer protection through the Kratom Consumer Protection Act (KCPA) or similar legislation. Common provisions include prohibiting sales to individuals younger than 21 years, requiring product labeling with accurate ingredient information and health warnings, and prohibiting synthetic kratom products. States including Colorado, Georgia, South Carolina, and Virginia have adopted comprehensive laws requiring manufacturer registration, potency limits for active alkaloids (mitragynine and 7-hydroxymitragynine), and penalties for violations [17].

In addition to promoting consumer safety, these regulations seek to address the limited oversight of kratom products, which are not currently regulated by the United States Food and Drug Administration (FDA). While some states, such as Nebraska, have proposed classifying kratom as a controlled substance, most have adopted harm-reduction approaches that include age restrictions, secure retail practices (e.g., behind-the-counter storage), and civil or criminal penalties for noncompliance [17]. These legislative approaches reflect concerns regarding unregulated kratom products and their potential contribution to adverse health events, particularly among youth and other vulnerable populations, while also acknowledging the growing use of kratom for self-management of pain, anxiety, and opioid withdrawal symptoms [3,4]. This evolving policy landscape highlights the need for additional research into kratom's pharmacology, therapeutic potential, and public health impact [4].

Case studies and epidemiology

Case reports demonstrate that kratom toxicity can occur following high daily intake and use over days to weeks, particularly in the setting of co-ingestion with substances such as alcohol, opioids, and psychiatric medications, which are major contributors to toxicity risk [6,18]. Epidemiologic trends show increasing kratom exposure, with associated fatalities frequently involving co-ingested substances such as alcohol, marijuana, benzodiazepines, amphetamines, and cocaine [18,19]. This pattern is consistent with forensic toxicology data demonstrating that mitragynine is most often detected with co-ingestants in fatal overdose cases, limiting the ability to attribute causation to kratom alone [10,18,20]. Centers for Disease Control and Prevention (CDC) toxicology monitoring identified the presence of kratom in toxicology testing for unintentional overdose deaths across 27 states between July 2017 and December 2017. In most cases, one or more co-ingestants were also identified, preventing kratom from being reliably established as the primary cause of death [9]. Individuals with a history of substance use disorders are particularly vulnerable to kratom-related adverse effects [6]. These findings reiterate the need for targeted monitoring, regulation, and education regarding kratom use, particularly among at-risk populations. Comprehensive public health surveillance and epidemiologic research remain essential to inform evidence-based clinical practice and public health policy [4].

This pattern is consistent with recent case series data. A 2025 review of 95 kratom-associated acute toxicity cases reported presentations including respiratory depression, seizures, arrhythmias, hepatotoxicity, and altered mental status [21]. Another illustration of kratom's clinical complexity is the case of a young male who presented in critical condition with tachycardia, hypotension, pinpoint pupils, and severe respiratory distress. He did not respond to naloxone administered by Emergency Medical Services (EMS) and required intubation and admission to the intensive care unit (ICU). Routine urine drug screening and blood alcohol testing were negative. Laboratory studies demonstrated elevated liver enzymes, blood urea nitrogen, creatinine, troponin, amylase, lipase, and lactic acid. A computed tomography (CT) scan demonstrated cholestasis without evidence of cholecystitis or cholelithiasis. Subsequent history revealed that the patient had been taking a supplement purchased online to increase stamina. Subsequent urine testing demonstrated 7-hydroxymitragynine concentrations exceeding 500 ng/mL, which, together with the clinical presentation and laboratory findings, was considered consistent with kratom toxicity [22]. He remained in the ICU for two weeks, requiring hemodynamic stabilization with intravenous fluid resuscitation and a norepinephrine infusion before his neurologic examination, vital signs, and laboratory values normalized. He was subsequently extubated and discharged to an acute rehabilitation facility. Management was primarily supportive, and the original authors described the response to naloxone as "partial at best" [22].

A 2023 case report describes a woman who became unresponsive at home and was transported to the emergency department by her husband. She responded to naloxone administration but denied opioid use, and routine urine drug screening was negative for opioids. After further questioning, she reported purchasing kratom tablets and a bottle of kratom liquid extract from a gas station earlier that day. During emergency department observation following naloxone administration, her oxygen saturation declined and miosis recurred, prompting consultation with Poison Control. Poison Control advised that patients with suspected kratom toxicity may be at risk for recurrent respiratory depression and may require additional naloxone administration and up to 24 hours of observation [13].

The variable response to naloxone is consistent with recent preclinical evidence suggesting that responsiveness depends on which alkaloid predominates in the consumed product and highlights the diagnostic and management challenges associated with kratom toxicity. Because kratom alkaloids are not detected by routine opioid screening and may have prolonged pharmacologic effects, patients may remain at risk for delayed recurrent respiratory depression despite initial clinical improvement [14,15]. These reports support careful observation following naloxone administration, consideration of repeat naloxone dosing when clinically indicated, and patient education regarding the potential for delayed respiratory compromise.

Clinical challenges

Clinical challenges may arise when symptom resolution after initial naloxone administration is interpreted as recovery, particularly in patients with opioid-like presentations whose routine opioid immunoassay screening is negative [13]. This may provide false reassurance and contribute to premature discharge or inadequate observation because mitragynine and 7-hydroxymitragynine have substantially longer durations of action than naloxone. Human pharmacokinetic data indicate that mitragynine has a half-life of up to 68 hours, which far exceeds naloxone's 30-90-minute duration of action [12,23]. Reported cases have described recurrent respiratory depression following initial improvement after naloxone administration, highlighting the importance of continued clinical observation in selected patients [13].

Clinicians should maintain a high index of suspicion for kratom toxicity when patients present with opioid-like features despite negative routine opioid immunoassay screening, recognizing that standard urine drug screens do not detect kratom alkaloids and may not identify all synthetic opioids or other co-ingestants [13]. Observation periods and disposition decisions should therefore be individualized based on the patient's clinical course rather than the initial response to naloxone alone. When patients elect to leave against medical advice, naloxone kits should be provided together with clear instructions for use and appropriate follow-up recommendations to reduce post-discharge complications.

The importance of careful observation becomes even greater in cases involving co-ingestion. Alcohol, opioids, and benzodiazepines are frequently identified in kratom-related fatalities, as they may potentiate kratom's sedative and respiratory-depressant effects [8,9]. Alcohol, as a central nervous system depressant, may increase the risk of respiratory compromise and cardiovascular instability when combined with kratom [6]. Similarly, concomitant opioid use may further increase the likelihood of respiratory depression through additive μ-opioid receptor activation [1,2]. Benzodiazepines may also enhance respiratory depression when co-administered with kratom, increasing the risk of severe toxicity [6].

These pharmacodynamic interactions not only increase clinical risk but also highlight an emerging area of clinical knowledge. As kratom use becomes more prevalent in Western countries, its pharmacologic properties, potential for delayed toxicity, and risks associated with polysubstance use may not yet be routinely emphasized during clinical training. This may contribute to under-recognition, delayed diagnosis, and missed opportunities for patient counseling. As kratom use continues to increase, there is a growing need to equip clinicians with evidence-based knowledge to support clinical decision-making, optimize patient outcomes, and inform public health strategies [3,4].

Clinical considerations in suspected kratom toxicity

Available case reports and toxicologic studies suggest that kratom-associated toxicity may present with opioid-like signs, including respiratory depression, miosis, and altered mental status, even when routine opioid immunoassay screening is negative because kratom alkaloids are not detected by these assays [9,13,14]. In such cases, naloxone administration may produce partial or transient clinical improvement, reflecting differences in the pharmacologic activity and duration of kratom alkaloids, particularly 7-hydroxymitragynine, relative to naloxone [2,12,15].

Several reports describe recurrent hypoventilation or declining oxygen saturation following an initial response to naloxone, raising concern for delayed respiratory compromise [13,14]. These reports support careful reassessment after initial clinical improvement, particularly in patients with significant respiratory depression, suspected high-dose kratom exposure, or co-ingestion of other central nervous system depressants [8,9,18].

Because mitragynine and its metabolites are not detected by routine opioid immunoassays, clinicians should consider kratom toxicity in patients with opioid-like presentations despite negative routine opioid immunoassay results [9,13,20]. Management remains primarily supportive and should include airway protection, ventilatory support when indicated, repeat naloxone administration based on the patient's clinical response, and observation guided by the patient's overall clinical course rather than the initial response to naloxone alone [14,21].

Although the available evidence is derived primarily from case reports and retrospective studies, the consistency of reported delayed respiratory effects supports careful monitoring and individualized disposition decisions for patients with suspected kratom toxicity [6,10,21].

Discussion

The literature reviewed throughout this paper demonstrates the complex and often paradoxical nature of kratom use. While traditional forms in Southeast Asia have been consumed for generations with relatively few reported serious adverse effects, patterns of use in Western countries are characterized by higher doses, concentrated extracts, and more frequent co-ingestion with other substances, introducing additional toxicologic and regulatory considerations [1,3]. Epidemiologic data indicate that most kratom-associated fatalities involve one or more co-ingestants, emphasizing the importance of comprehensive clinical evaluation, assessment for co-ingestants, and individualized risk assessment [8,9,18].

Pharmacologically, kratom exhibits partial μ-opioid receptor agonism together with serotonergic and adrenergic activity, contributing to its complex, dose-dependent clinical profile [1,2]. At lower doses, stimulant-like effects predominate, whereas higher doses are associated with sedation, respiratory depression, and, in rare cases, hepatotoxicity [1,6]. These highly variable effects, together with the absence of standardized laboratory testing for kratom alkaloids, may complicate diagnosis and delay treatment [12,19]. Human pharmacokinetic studies demonstrate that mitragynine has a substantially longer half-life than naloxone; however, pharmacokinetic differences alone do not establish prolonged clinical toxicity. Nevertheless, reported cases describing recurrent respiratory depression after initial naloxone response suggest that careful clinical observation may be warranted in selected patients with suspected kratom toxicity [12,14]. Experimental studies further demonstrate that 7-hydroxymitragynine is an active metabolite of mitragynine with substantially greater μ-opioid receptor efficacy, contributing to its opioid-like effects [24].

The KCPA has been adopted in numerous states and represents meaningful progress toward improving product labeling, standardizing alkaloid concentrations, and enhancing consumer safety [17]. However, implementation remains inconsistent across jurisdictions, and kratom products continue to vary considerably in composition and potency. Additional research is needed to better characterize product variability, establish standardized analytical detection methods, and evaluate how these differences influence clinical toxicity and patient outcomes [4,17].

Despite growing interest in kratom, important knowledge gaps remain. Most available evidence is derived from case reports, case series, retrospective studies, and preclinical investigations, limiting the ability to establish causality or develop evidence-based management guidelines. There are currently no prospective human studies specifically evaluating the relationship between kratom alkaloid pharmacokinetics and clinical response to naloxone. Future research should focus on prospective clinical studies, standardized toxicologic testing, and pharmacokinetic-pharmacodynamic investigations to clarify the relationship between kratom exposure, toxicity, and optimal clinical management. Concurrent efforts to improve clinician education and public awareness will be essential as kratom use continues to expand [3,4].

This narrative review has several limitations. Because it was not conducted as a systematic review, no formal inclusion or exclusion criteria or risk-of-bias assessment were applied. The available literature is composed primarily of case reports, retrospective studies, and preclinical investigations, limiting the ability to establish causality or develop evidence-based management recommendations. In addition, variability in kratom formulations and frequent polysubstance exposure make comparisons across published studies challenging.

## Conclusions

Kratom-associated toxicity represents an emerging challenge in acute care because it may present with opioid-like signs despite negative routine opioid immunoassay screening and may respond incompletely or transiently to naloxone. Reported cases describe delayed or recurrent respiratory depression, particularly following high-dose exposure or polysubstance use. Clinicians should maintain a high index of suspicion for kratom toxicity, provide supportive care, administer repeat naloxone when clinically indicated, and base observation and disposition decisions on the patient's clinical course rather than the initial response to naloxone alone.

Current evidence is derived primarily from case reports, retrospective studies, and preclinical investigations, highlighting the need for prospective human studies to better define optimal diagnostic and management strategies. As kratom use continues to increase, improved clinician awareness, continued research, and evidence-based regulatory approaches will be important for enhancing patient safety and informing future clinical practice.
